# The mediating effect of the need for cognition between aesthetic experiences and aesthetic competence in art

**DOI:** 10.1038/s41598-024-53957-6

**Published:** 2024-02-10

**Authors:** Agata H. Świątek, Małgorzata Szcześniak, Michał Stempień, Karolina Wojtkowiak, Marianna Chmiel

**Affiliations:** Faculty of Social Sciences, Institute of Psychology, University of Szczecin, Szczecin, Poland

**Keywords:** Aesthetic experience, Aesthetic competence, Need for cognition, Mediation, Human behaviour, Cognitive control

## Abstract

Although the role of aesthetics and aesthetic education in everyday life was discussed as early as the ancient philosophers, the psychological mechanisms shaping the aesthetic quotient have hardly been investigated by empirical studies. The aim of this study was to examine the direct relationship between experience and aesthetic competence, and the mediating role of need for cognition. The study involved 201 Polish adults, aged 18 to 76 (*M* = 26.40; *SD* = 11.89), 65% of whom were women. The respondents completed anonymous questionnaires on an online platform. The surveys included a metric, the Aesthetic Competence Scale (ACS), the Aesthetic Experience Questionnaire (AEQ) and the Need for Cognition Scale (NCS). A positive correlation coefficient was obtained between all three variables studied, with need for cognition acting as a mediator in the relationship between experience and aesthetic competence. The findings indicate that individuals reporting intense aesthetic experiences have a higher aesthetic competence if this relationship is mediated by a high need for cognitive effort.

## Introduction

Dutton^1^^, p. 273^ wrote that experiencing art is a natural, innate human trait and referred to Kant’s words that experiencing art is a practice of contemplation. It seems that exposure to art and the cognitive and emotional engagement in its reception fosters the development of aesthetic competence, which is a manifestation of the aesthetic quotient^2,3^. It is believed that looking at art is interesting and rewarding for audiences, regardless of their aesthetic preferences^4^. Art knowledge is known to moderate cognitive judgments, specifically in the sense that experts find abstract paintings more interesting and less confusing than non-experts^5^. In addition, the intensity of attention and effort, first-order visual processing, as well as arousal and sustained attention, in response to a work of art, are stronger in those with more artistic experience^4^.

However, is the mere multiplication of aesthetic experience enough to develop a high aesthetic competence? What role does the “thirst” for knowledge play in this process? This question prompted arguably the first study that used a mediation model to explain the role of cognitive effort in developing the aesthetic quotient.

## Literature review

In their work, Leder and Nadal^6^ provide a history of the rise, fading and re-emergence of psychologists’ interest in aesthetics. Pelowski et al.^7^ reviewed a selection of models explaining the process of viewing art. The discussion included the observation that insight, altered perception, harmony and aesthetic sensations are still insufficiently explored. Therefore, this area of research is not entirely new, but it should be noted that the empirical operationalization of key concepts, such as “aesthetic experience”^8^ and “aesthetic quotient”^3^ has been relatively recent.

### Aesthetic experiences

As pointed out by D’Olimpio^9^^, p. 242^, “Aesthetic experiences are dynamic and complex, and therefore notoriously difficult to pin down.” Leder et al.^10^ claim that aesthetic experiences are not only aesthetic emotions but also cognitive states that are expressed in judgments about aesthetic preferences. According to Chatterjee and Vartanian^11^, aesthetic experience can be described by a three-way model. In their view, aesthetic experiences emerge from the interaction between the neural system for emotion and evaluation, the sensory-motor system, and from the awareness of meaning. Redies^12^ notes that aesthetic experience remains poorly defined and poorly restricted as an object of empirical research. The author uses it as a general term—the intense feeling of pleasure that can be experienced when viewing beautiful stimuli. In developing their method of measuring aesthetic experience (AEQ) (such as, the perception of the details of a work of art or the emotions experienced), Wanzer et al.^8^ recognize flow as a particular state of mind^13^. It is also known that aesthetic experience goes beyond merely perceiving and experiencing beauty, as it is only one of the many dimensions of aesthetic assessment. To acknowledge that beauty is just one aspect of encounters with art and not a necessary one for aesthetic experiences^14^.

The ability to experience contact with art is underpinned by a specific, personal vulnerability. Research suggests that people differ in their visual and musical aesthetic sensibilities^15^. Brisson and Bianchi^16^^, p. 259^ write about aesthetic disposition, understood as “the propensity to prioritize form over function and to approach any object as potentially valuable from an aesthetic standpoint.” Corradi et al.^17^^, p. 1^ describe artistic sensitivity as the “ability to recognize and appreciate beauty and compositional excellence, and to judge artistic merit according to standards of aesthetic value.” People who have high aesthetic sensitivity are more sensitive to the aesthetic properties of the stimuli^17–19^. Significant relationships have been observed between a high capacity for aesthetic “emotion contagion” and a declared fondness for art, and stronger aesthetic experiences, such as being moved, valence, and interest^20^. In a study involving a Taiwanese sample, it was shown that being a woman, graduating with a degree in art or design, or being a religious person, is associated with a richer aesthetic life and that aesthetic experience promotes mature ego identity development^21^.

In their article, Christensen et al.^22^ reflected on the ways of conducting empirical research in the field of aesthetics and divided them into two groups. One involves an attitude toward evaluative judgment about an artwork. The second concerns how the work of art aroused thoughts and feelings conducive to understanding art’s various impacts. Among them, they mentioned research regarding curiosity (the desire to acquire knowledge), having insight or an “aha” moment (new understanding), and experiencing profound emotions as sublime (transformation of the way of thinking). Therefore, it can be assumed that the possible or potential goal of aesthetic experience is psychological change. This seems consistent with Pelowski et al.^7^, who proposed a model combining the previous knowledge about human interaction with art. In their Vienna Integrated Model of Art Perception, the culmination of the process is the level of self-awareness and metacognitive assessment. Through aesthetic experiences, an individual develops aesthetic competences and, consequently, deepens their ability to cognitively transform themself.

However, mere susceptibility to and the intensity of aesthetic experiences does not necessarily correspond to having high artistic competence. Art and music are a type of code, a language^23^, so a full understanding of aesthetic objects requires the ability to contextualize. The potential for experiencing strong aesthetic sensations does not necessarily imply the ability to estimate the artistic value of a work of art, the possession of specialized knowledge or a long history of interaction with art, although it probably helps in acquiring them.

### Aesthetic competence

Intelligence is undoubtedly one of the most popular areas of interest for psychologists. For example, Legg and Hutter^24^^, p. 22^ analyzed 71 general definitions of intelligence and proposed their own: “Intelligence measures an agent’s ability to achieve goals in a wide range of environments.” Can this concept be narrowed down to a specific type of aesthetic knowledge and skills?

In psychology, there are various concepts of aesthetic intelligence. A fairly reliable chronological review of the ways in which aesthetic abilities are addressed was made by Dan et al.^2^. Some are complex and include a number of components, such as the Aesthetic Quotient concept developed by Myszkowski and Zenasni^3^. There are also concepts that are almost absent from the psychological literature. For example, Ferrucci^25^, a psychosynthesis therapist, believes that people have a universal capacity for appreciating beauty, termed aesthetic intelligence^26^, which can be nurtured and improved. Therefore, the results of integrating beauty is an idea (“inner transformation”) not so distant from the final stages of the art perception described by Pelowski et al.^7^ in a systematic way.

Moreover, it can be considered debatable as to how much to emphasize the role of beauty in aesthetic experience and theories centered around beauty when it comes to aesthetic intelligence. Is the concentration on beauty limiting and overlooking other—possibly more relevant—aspects of the aesthetic understanding of art? We acknowledge that beauty is only one of the different components that constitute an aesthetic experience^8^. According to us, it is possible to differentiate between the other aesthetic qualities of an object and its beauty, and we believe that it is possible to have an aesthetic experience with an object that we do not necessarily perceive as beautiful. For example, the paintings of Beksiński (born 1929, died 2005) can hardly be called “beautiful”. The ugliness (horror, decay, and visions of destruction) is determined by the content, not the way the elements are composed and the quality of the artist's skills. Their content is a denial of the ancient, Platonic understanding of Beauty, related to Good and Truth, but at the same time, the works of this artist may be recognized and appreciated due to their complexity, ambiguity, and the artist's attention to detail.

As with other intelligence quotient concepts and their domains, the reliable measurement of aesthetic quotient levels poses a considerable challenge. Dan et al.^2^^, p. 5^ write: “People’s abilities of artistic perception, appreciation, and creation were chosen as the manifestation of their AQ.” They propose that the aesthetic quotient should be measured as a separate assessment of the intensity of aesthetic competence, in various areas of art. The conventional understanding of the word “competence” in Polish^27^, is, “the extent of someone’s knowledge, skills and experience,” so it includes not only what is innate but also what is acquired over time. Within the scale developed by Dan et al.^2^, art knowledge, sensitivity (emotional responsiveness to art), understanding (reflection on the work) and the ability to evaluate the quality of a work of art are not viewed as separate components of aesthetic intelligence, but rather as a manifestation of competence in evaluate artworks a particular domain. The overall score is calculated by adding up the results for the different art domains^26^.

Susceptibility to aesthetic experiences (and their intensity) would, therefore, be a component, not a full manifestation of AQ. The authors would like to mention that the degree of convergent validity of the two concepts puzzled us during a broader study aimed at adapting the Aesthetic Experience Questionnaire^28^ to Polish cultural conditions. We expected high correlations between aesthetic experiences and aesthetic competence. At the time, we assumed that aesthetic experiences, as overlapping with (included in) aesthetic quotient, should be strongly correlated with ACS, but the correlations were moderate. The findings suggested partial independence of the two constructs. This is consistent with reports indicating that individuals with expert knowledge show a distinct pattern of attenuated emotional and value reactions to the works they see^6^, although, as noted earlier, experts display higher cognitive engagement^4,5^. The authors assume that sensitivity to aesthetic sensations and the accumulation of such experiences are conducive to the development of competence in art, but in order to achieve a high aesthetic quotient and analyze art from angles other than personal preference or sensory pleasure, something else is needed.

### Need for cognition

Works of art are sometimes ambiguous, unclear and complex, both in terms of their form and the message conveyed by the artist. The reception of artworks requires reflection and focused engagement. Controlled cognitive processing of an artwork is identified as an important aspect of aesthetic experience^29^. The question is, what makes some people prefer ambiguous art? It is known, for example, that art experts are more likely to enjoy negative or even provocative works^6^.

Presumably, the quality that encourages in-depth analysis of works of art is the need for cognition. Wu et al.^30^, characterize it as an individual’s predisposition to engage in and derive pleasure from thinking. Psychologists’ interest in this issue has quite a long tradition – a publication that analyzes more than 100 articles presenting the results of research on individual differences in the need for cognition^31,32^ has been cited 3549 times to date [Google Scholar, as of 04.04.2023], while Petty et al.^33^ noted that more than 1000 articles cited the original article or presented shortened versions of the NCS scale. A high need to explore and engage in intellectual activity is predictive of behaviors related to drives, goals, and allocation of attention and is also correlated with fluid aspects of intelligence and traits, such as openness to experience and emotional stability^34^. Individuals with a high need for cognition enjoy actively thinking about and understanding the world, prefer complex tasks and want to understand causes, and gather as much information as possible before drawing conclusions^35^.

Justification as to why one can feel aesthetic appreciation when viewing an ambiguous image and why cognitive engagement in the reception of art can be pleasurable is provided by neuroaesthetic research. Sarasso et al.^36^ report that aesthetic appreciation is correlated with the activation of dopaminergic circuits associated with reward. They explain that aesthetic emotions are a response to behavioral inhibition (physical activity) and promote the enhancement of perceptual processing in the sensory cortices, which are responsible for optimizing learning. The researchers^36^^, p. 724^ suggest that “aesthetic appreciation may represent a hedonic feedback over learning progresses, motivating the individual to inhibit motor routines to seek further knowledge acquisition.” Individuals with a high need for cognition may find engagement in viewing works of art more satisfying and rewarding than those with only low levels of this trait. A person who, in addition to being susceptible to aesthetic experience, also tends to enjoy immersing themselves in complex issues, may find it easier to develop high aesthetic competence.

### Hypotheses

The positive relationship between aesthetic experiences and the associated competency was observed as far back as antiquity. Plato considered aesthetic education to be one of the key components in the education and upbringing of a free man^37^. Indirectly, data indicates that aesthetic competence is acquired through education and the accumulation of aesthetic experiences. Empirical evidence of a link between frequent exposure to art, acquisition of art knowledge and high aesthetic competence is indicated by the research of Dan et al.^2^. They observed that art students achieved higher levels of AQ than literature students. Indirectly, higher aesthetic competence in those with more experience with art is also demonstrated by a study by Mullenix and Robinet^38^. The participants in the study rated a set of abstract paintings for liking and understanding. Expert knowledge was found to be positively correlated with the level of understanding of the paintings and with faster decision-making (suggesting higher aesthetic competence) but not with liking. Accordingly, this study assumed that:

#### H1

Aesthetic experience is positively related to aesthetic competence.

According to D’Olimpio^9^, aesthetic experiences have a phenomenological quality that includes being absorbed, focused, and open. In order for aesthetic experience to be enriching, one needs to be interested in exploring a new object and to show curiosity, coupled with excitement and anticipation of what can happen by engaging with and perceiving the object. The desire for exploration associated with exposure to art, which the researcher describes from a pedagogical perspective, seems consistent with the psychological understanding of the need for cognition.

Data suggests that a high need for cognition is correlated with three of the five dimensions of flow: focused concentration, sense of control, and curiosity^39^. If flow is recognized as a potential component of aesthetic experience^8^, then declaring a stronger aesthetic experience is more likely to be positively associated with a higher need for cognition. In research on the construction of the Aesthetic Experience Questionnaire, Wanzer and her team^8^ verified the correlations between aesthetic experiences and measures of openness to experience, inspiration, curiosity, and exploration. Further, the research on the Polish adaptation of this scale revealed that “[t]he inclination for analytical observation of reality may encourage greater engagement in contemplation of art”^28^^, p. 04^, as aesthetic experience is correlated with need for cognition. Therefore, the following hypothesis was formed:

#### H2

Aesthetic experience is positively related to need for cognition.

Kowalik^40^ writes from the perspective of a practicing psychologist and a scientist that both creating and receiving art are specific ways of learning about reality. He describes at least three types of cognitive void (metaphysical, epistemic and existential), which people try to limit, to fill selectively or globally, with various types of art. In general, about all types of cognitive void experienced by people, he metaphorically wrote that it is a “blank spot” in the space already known, which needs to be filled. And so, successively, the metaphysical void is, “the feeling of the non-existence of something that we would like to exist”^40^^, p. 399^ and the psychological function of the metaphysical void is to organize the ontological space being explored and to enable it to be enriched with fictitious elements. An epistemic void is a “lack of knowledge about something that we suppose may exist (or indeed does exist), but for some reason is inaccessible to our cognition”^40^^, p. 399^. Filling the epistemic space is mainly the responsibility of scientists and individuals who feel individual deficiencies in epistemic knowledge and try to fill this kind of void by referring to individual experience. The third type of void is an existential void, which differs from the previous ones in that it concerns introspective awareness, the feeling of not knowing oneself (lack of knowledge about one’s inner life, surprise with one’s own behaviors, differences between the self and others, etc.). Having a strong need for cognition promotes the integration of previous art knowledge and experience and prompts the viewer to reduce feelings of uncertainty. Since intellectual engagement with art is self-rewarding^36^, presumably the repetitive nature of this process will translate into increased aesthetic competence. The following assumption was therefore made:

#### H3

A high need for cognition is positively related to aesthetic competence.

With reference to the data cited in the literature review, it was assumed that individuals reporting higher levels of aesthetic experience would exhibit a higher aesthetic quotient if they had a high need to engage in intellectually challenging tasks. In addition, putting need for cognition in the role of mediator between aesthetic experience and aesthetic quotient would be in line with data on other thematic studies in which the NCF acted as a mediator^41–43^ or moderator^44–46^. For that reason, the expectation was as follows:

#### H4

Need for cognition plays a mediating role in the relationship between aesthetic experience and aesthetic competence.

## Tools

### Aesthetic Competence Scale

The Aesthetic Competence Scale (ACS), created by Dan et al.^2^, is used to measure the aesthetic quotient in relation to four selected art areas. It refers to the extent to which the recipient can assess the quality of artworks but it does not measure proficiency in artistic skills. The Polish version of the tool was used in this study^26^. The scale consists of 20 statements, with five each being part of the competence factor in music, visual arts, literature, and film. The respondents expressed their opinions using a five-point Likert scale (1 = “strongly disagree,” 5 = “strongly agree”). The sum of the scores from all subscales indicates the overall level of aesthetic competence in the arts. The higher the overall score, the greater the aesthetic competence of the respondent. In the present study, the individual dimensions, as well as the overall score, had high reliability coefficients: music (α = 0.90), visual arts (α = 0.89), literature (α = 0.91), film (α = 0.92) and the overall aesthetic competence score (α = 0.96).

### Need for Cognition Scale

The Need for Cognition Scale (NCS) is a one-dimensional tool to assess the propensity to engage in and enjoy cognitively demanding tasks. The original version of the tool, created by Cacioppo and Petty^31,32^, was adapted to Polish conditions by Matusz et al.^47^. The questionnaire consists of 36 statements rated by respondents on a five-point Likert scale (from 1—“strongly disagree” to 5—“strongly agree”). The points obtained by the respondent are summed up to obtain the overall score. A high overall score indicates a high need for cognitive engagement. In the present study, Cronbach’s alpha coefficient was α = 0.86.

### Aesthetic Experience Questionnaire

The Aesthetic Experience Questionnaire (AEQ), created by Wanzer et al.^8^, assesses the intensity of experiences associated with exposure to art. As intended by the authors, the scale applies primarily to the visual arts (e.g., painting, drawing, sculpture, and printmaking), but can also be applied to other areas of art. The study used the Polish version of the AEQ^28^. Like the original, the Polish version contains 22 statements grouped into six aspects of aesthetic experience: the emotional, cultural, perceptual and understanding dimensions, conditions conducive to flow, and experience of the flow state. Survey participants check the degree to which they agree with each statement they read on a 7-point Likert scale (where 1 means “strongly disagree” and 7 “strongly agree”). The points are added up within each of the subscales. An overall score is also available. In this study, the reliability coefficient proved satisfactory for all dimensions of the questionnaire: emotional (α = 0.91), cultural (α = 0.86), perceptual (α = 0.88), understanding (α = 0.86), conditions conducive to flow (α = 0.88), experience of flow (α = 0.91), as well as for the overall score (α = 0.96).

### Study group and procedure

The study involved 201 adult Poles, with 65% of the respondents being women. The participants’ ages ranged from 18 to 76 (*M* = 26.40; *SD* = 11.89). The participants were asked about their level of education. The largest group was currently studying (48%), followed by individuals with higher education (33%), with significantly fewer respondents declaring that they had obtained secondary education (9%), vocational technical education (6%) or primary education (3%). The fewest respondents reported vocational education (1%).

A number of studies have focused on the differences between experts and non-experts (novices or those with particularly low aesthetic scores). We wanted to understand the general direction of the relationship between aesthetic experience and aesthetic quotient, so we decided not to divide the study sample by expertise. We tried to exclude individuals who were completely uninvolved or disinterested in art, as well as those with a very high level of expertise (e.g. several years of work in the arts) from participating in the study and reached out mainly to those declaring average interest and/or amateur involvement in the arts.

Non-random sampling (accidental and snowball sampling) was used. The respondents were made aware of the purpose of the study, its structure, potential duration, the possibility of discontinuing participation at any time and about the anonymity and confidentiality of individual results. All participants provided informed, written consent. The study was based on an online, asynchronous, one-time questionnaire. The research process took place under Approval No 6/2022 of the Research Ethics Committee of the Institute of Psychology at the University of Szczecin. All methods were carried out in accordance with the Declaration of Helsinki.

### Statistical analysis

Analysis of the data obtained was carried out using IBM SPSS Statistics 23.0 and the PROCESS macro for SPSS (version 3.2)^48^. The data distribution was examined by means of the Shapiro–Wilk test^49,50^. Since such normal distribution values were not obtained in our study, a Spearman’s Rho correlation was performed to verify the association between aesthetic experience, need for cognition, and aesthetic competence.

A linear regression model was used to (1) detect the degree of multicollinearity, (2) locate prospective outliers and (3) account for potential confounders, and to scan whether they were impacting the existent relationship between the independent (aesthetic experience) and dependent (aesthetic competence) variables. First, because we counted that the explanatory variables (aesthetic experience and need for cognition) could share considerable variance, we scrutinized whether there would be a multicollinearity problem. To diagnose collinearity, we used overall measures (the tolerance statistics and the variance inflation factors, VIF)^51^. Tolerance lower than 0.1 to 0.2 and VIF larger than 5 to 10^52^ were assumed as indicators of a serious multicollinearity problem. Next, we computed Mahalanobis’ distance (*p* < 0.001) and Cook’s distance (greater than 1) to screen for the presence of multivariate outliers^53^. Finally, the respondents’gender, age and education were inserted to control for their potential impact on the relationship between the aesthetic experience and aesthetic competence. Indeed, there is some empirical evidence, although very limited, that sex has an effect when it comes to art. Some subtle differences between women and men can be noticed with respect to aesthetics^54^. Age has also been found important for the development of aesthetic experience and judgment^55^. Moreover, aesthetic experience has been considered as a part of the social world^56^ and cultural experience^56,57^, thus being possibly associated with the level of education. All three potential confounders (gender, age, and education) were entered at Step 1. All predictors of aesthetic competence were entered at Step 2. Information about multicollinearity, outliers, and confounding factors is provided in the Supplementary materials.

## Results

### Descriptive statistics and correlations

Descriptive statistics for the mean, standard deviation, values of the Shapiro–Wilk test, and correlations for each variable are shown in Table 1. Since the Shapiro–Wilk tests for normality showed that only the NCS presented a non-significant *p* value, Spearman’s Rho correlations were calculated for each variable considered in the study (aesthetic experience, aesthetic competence, need for cognition).

**Table 1:** Mean, standard deviation (SD), Shapiro–Wilk test, and correlations of the study variables (N = 201).

	*M*	*SD*	Shapiro–Wilk test	1	2	3
1. AEQ	4.10	32.11	W = 0.97; *p* = 0.001***	1		
2 ACS	3.76	12.99	W = 0.96; *p* = 0.001***	0.52***	1	
3. NCS	3.58	0.50	W = 0.98; *p* = 0.053 (ns)	0.36***	0.43***	1

***p < 0.001; ns: non-significant value; AEQ: Aesthetic Experience Questionnaire; ACS: Aesthetic Competence Scale; NCS: Need for Cognition Scale.

### Mediating effect of need for cognition

**Figure 1:**
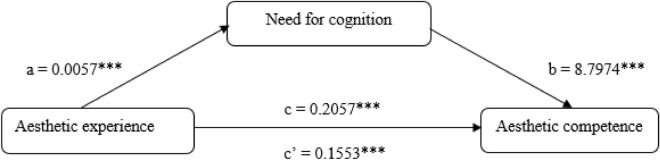
Theoretical model of the role of need for cognition in the relationship between aesthetic experience and aesthetic competence. *p < 0.05; ***p < 0.001.

In order to verify the hypothesis that need for cognition (H4) plays a mediating role in the relationship between aesthetic experience and aesthetic competence, Model 4, a single-mediator mediation model, was chosen (Fig. 1). The results of the analysis indicate that aesthetic experience is an explanatory variable for need for cognition. This means that a high intensity of aesthetic experience is predictive of a high need for cognition (path a: β = 0.0057; p < 0.001). In addition, need for cognition was found to be predictive of the level of aesthetic competence (path b: β = 8.7974; p < 0.001).

In the next step, the occurrence of mediation was verified using the 5,000 bootstrap samples method, assuming a 95% confidence interval without zero. A statistically significant indirect effect (0.0504) with B(SE) = 0.0137 was observed for the impact of aesthetic experience on the level of aesthetic competence, with need for cognition as a mediator (95% CI: 0.0262; 0.0792). The results indicate that β = 0.2057 at *p* < 0.001 (path c) decreases to β = 0.1553 at *p* < 0.001 (path c′), confirming the mediating role of the need for cognitive stimulation (H4).

## Discussion

The established hypotheses were confirmed by statistical analyses. The results obtained are consistent with reports by Dan et al.^2^ and Myszkowski and Zenasni^3^ stating that interaction with art and emotional and cognitive engagement in its reception promotes the development of aesthetic competence in appraising artworks. The results contribute new knowledge to the existing body of data in the literature. Arguably, this is the first mediated study to further clarify the role of the need for cognition in the formation of aesthetic competence. The need for cognition provides an opportunity to explore the impact of the arts on our senses and thinking and helps ensure that aesthetic experiences are collected and analyzed in a purposeful way, supporting the development of aesthetic competence.

From an evolutionary perspective, the ability to receive and create art can hardly be seen as adaptive for the species. Luty, citing Dutton^58^^, p. 31^, argues that art is a by-product of one or more other cognitive abilities. On the other hand, this “side effect” can improve an individual’s adaptability in everyday life. Although the arts seemingly do not have the power to directly improve people’s lives and, perhaps for this reason, remain a marginalized area of general psychological knowledge^38^, the information and new perspectives gained from viewing, listening to or practicing them can be used to better cope with the challenges of everyday life. It is known, for example, that creative artistic activity facilitates coping with emotions through the acquisition of new insights, self-development or distancing oneself and avoiding difficulties^59^, while listening to music not only regulates emotions, such as excitement but also serves a communal, bonding function^60^. By experiencing art and increasing aesthetic competence through the need for cognition, one builds sensitivity to details, a broader context and an understanding of cultural phenomena, and learns to be open to the non-obvious and ambiguous.

Finally, it should be added that it is important that this paper popularizes two relatively new psychological tools (ACS and AEQ) that have only been validated for Polish cultural conditions in recent months, so it simultaneously validates the psychometric properties of these tools and encourages other researchers to use them in their projects.

In addition, it is noteworthy that age proved to be a confounding variable, which suggests that aesthetic competence decreases with age. Younger people (high school graduates) may have higher competences in art, because knowledge about art and culture has been acquired during obligatory education or gained thanks to the Internet and its ease of access. In people in middle adulthood, engagement with arts (unless someone works or has a hobby in this field) is probably not a priority in this period of life, considering that middle adults’ developmental tasks require a lot of time, energy, and resources, which do not leave space for art.

## Limitations

As with any quantitative, online study, despite the care taken to ensure that the survey was a one-time event, that consent was obtained and that access to the link was terminated upon completion of the survey, there were limitations inherent in this form of data collection^61^. Doubts may be raised about the sample. Eighty percent of the participants had higher education or were studying at university and the mean age of the respondents was approx. 26. In order to relate the results to the general population, it is advisable to conduct the survey among a group of people with different educational backgrounds and a higher modal value for age, and to compare the results based on the expertise in each art area.

A limitation of the authors’ study is the fact that art-related scales were used. This means that we cannot extend the conclusions from our research to aesthetic competences in general, i.e. non-artistic ones. In fact, any stimulus can be perceived as aesthetic, and aesthetics and art are two separate theoretical constructs. It is possible to talk about and research aesthetics without referring to human or non-human products (artworks), although it is difficult to talk about art apart from aesthetic values.

### Supplementary Information

Supplementary Information.

## Data Availability

All data have been made publicly available at osf and can be accessed at https://osf.io/xr2de/?view_only=b960889013a343b598d06e7cc8b73ef8.
