# Supplementary material for: The mediating effect of the need for cognition between aesthetic experiences and aesthetic competence in art
Source: Sci Rep. 2024 Feb 10;14:3408. doi: 10.1038/s41598-024-53957-6 (PMC10858861; doi:10.1038/s41598-024-53957-6)
Supplement: Supplementary file 1 — Supplementary Information. [file 41598_2024_53957_MOESM1_ESM.docx]

**SUPPLEMENTARY MATERIAL**

*Multicollinearity, Outliers, and Confounders*

The Variance Inflation Factor values ranged well below 10 (between 1.023 and 1.260) and the lowest tolerance was 0.793 (far beyond 0.2), indicating no presence of multicollinearity in the data. The Mahalanobis distance for multivariate outlier detection revealed a chi-squared value of less than 0.001 in only three of the 201 cases. The scatterplot of the standardized residuals against standardized predicted values (Supplementary Figure 1) confirmed the presence of three outliers.


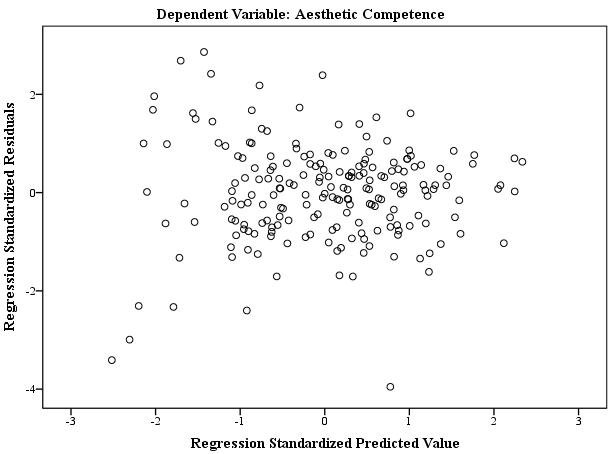


**Supplementary Figure 1.** Scatterplot of the standardized residuals against standardized predicted values.

Although both unstandardized and standardized residuals were not normally distributed (Shapiro-Wilk values for both were *p* = 0.001), removing these outliers was unnecessary since the statistics were very similar with or without them. Based on these outcomes, we assumed that they were not influential observations. Likewise, the Cook’s distance values (between 0.000 and 0.222) corroborated that the outliers were not problematic. Additionally, the diagonal line on the normal P-P Plot of regression standardized residual for dependent variable (Aesthetic Competence) indicate no substantial deviations from these variables (Supplementary Figure 2).


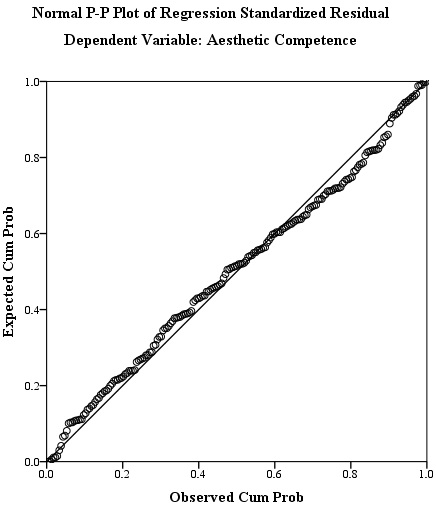


**Supplementary Figure 2.** Normal P-P Plots of Regression Standardized Residual of Aesthetic Competence

The linear regression model showed that two of gender categories, (β = −0.049, t = −0.856, *p*= 0.393), age (β = −0.186, t = −2.960, *p* = 0.003) and education (β = 0.086, t = 1.385, *p* = 0.168) explained only 1.7% of the variance (R^2^ = 0.017). However, age had a significant and negative effect, suggesting that aesthetic competence may decrease with age. Aesthetic experience (β = 0.394, t = 6.526, *p* = 0.001) and need for cognition (β = 0.363, t = 5.914, *p* = 0.001) predicted a significant amount of the variance (additional 37.2%; *F*(200,5) = 24.814, *p* = 0.001) even after controlling for the effects of confounders.
